# Multivariable prediction models for health care spending using machine learning: a protocol of a systematic review

**DOI:** 10.1186/s41512-022-00119-9

**Published:** 2022-03-24

**Authors:** Andrew W. Huang, Martin Haslberger, Neto Coulibaly, Omar Galárraga, Arman Oganisian, Lazaros Belbasis, Orestis A. Panagiotou

**Affiliations:** 1 Department of Health Services, Policy and Practice, Brown University School of Public Health, Rhode Island Providence, USA; 2 QUEST Center, Berlin Institute of Health, Charité–Universitätsmedizin Berlin, Berlin, Germany; 3 Department of Biostatistics, Brown University School of Public Health, Providence, Rhode Island USA; 4 Meta-Research Innovation Center Berlin, QUEST Center, Berlin Institute of Health, Charité–Universitätsmedizin Berlin, Berlin, Germany; 5 Center for Evidence Synthesis in Health, Brown University School of Public Health, Providence, Rhode Island USA

**Keywords:** Health care spending, Machine learning, Prediction, Systematic review

## Abstract

**Background:**

With rising cost pressures on health care systems, machine-learning (ML)-based algorithms are increasingly used to predict health care costs. Despite their potential advantages, the successful implementation of these methods could be undermined by biases introduced in the design, conduct, or analysis of studies seeking to develop and/or validate ML models. The utility of such models may also be negatively affected by poor reporting of these studies. In this systematic review, we aim to evaluate the reporting quality, methodological characteristics, and risk of bias of ML-based prediction models for individual-level health care spending.

**Methods:**

We will systematically search PubMed and Embase to identify studies developing, updating, or validating ML-based models to predict an individual’s health care spending for any medical condition, over any time period, and in any setting. We will exclude prediction models of aggregate-level health care spending, models used to infer causality, models using radiomics or speech parameters, models of non-clinically validated predictors (e.g., genomics), and cost-effectiveness analyses without predicting individual-level health care spending. We will extract data based on the Checklist for Critical Appraisal and Data Extraction for Systematic Reviews of Prediction Modeling Studies (CHARMS), previously published research, and relevant recommendations. We will assess the adherence of ML-based studies to the Transparent Reporting of a multivariable prediction model for Individual Prognosis Or Diagnosis (TRIPOD) statement and examine the inclusion of transparency and reproducibility indicators (e.g. statements on data sharing). To assess the risk of bias, we will apply the Prediction model Risk Of Bias Assessment Tool (PROBAST). Findings will be stratified by study design, ML methods used, population characteristics, and medical field.

**Discussion:**

Our systematic review will appraise the quality, reporting, and risk of bias of ML-based models for individualized health care cost prediction. This review will provide an overview of the available models and give insights into the strengths and limitations of using ML methods for the prediction of health spending.

## Background

Total health spending per capita has increased exponentially in nearly every country with concerning implications for the sustainability of global health financing systems, and by extension healthcare delivery systems [1, 2]. Numerous approaches have been implemented for constraining the growth of health spending, including capitated payments and other value-based insurance designs [3]. However, these approaches often rely upon the efficient allocation of resources based on predictions of future health spending. A common example is the risk adjustment of health plan payments which aims to reallocate funding towards plans with enrolled beneficiaries that are predicted to have higher than average annualized cost of care [4]. In this context, the efficiency of the health financing system is dependent on prediction models that can accurately estimate individual health spending, and the development of such models is a key topic in the field of health services research.

Regression-based techniques have been the most commonly used methods to predict health spending. However, these parametric approaches typically rely on strong assumptions about the true data generating mechanism and have difficulty with sparse or missing data [5, 6]. With recent advances in computation, machine learning (ML) techniques are being applied more frequently in prediction models for health spending. This includes, for example, the classification of patients into groups based on predicted spending amounts (“high-cost” or “high spenders”) or groups based on predicted changes in spending amounts over time (“cost bloomers”) [7, 8]. Compared to prediction models developed with parametric techniques, ML algorithms, such as random decision forests, neural networks, and penalized regression, have theoretical advantages and are generally considered more efficient, because they rely on fewer assumptions and have the ability to learn adaptively from the data [6, 9].

Despite their potential advantages, the successful implementation of ML methods to predict healthcare costs in routine settings could be undermined if their predictive performance is poor or leads to overly optimistic predictions. Various elements in the design, conduct, and analysis of ML models may introduce biases, including the lack of internal validation to prevent overfitting, unrepresentative sampling, or unaccounted missing data. The utility of these models may be also adversely affected by poor or inadequate reporting of the studies in the increasing body of literature through which they are disseminated to potential users including payers, health systems, and also individuals.

Previous systematic reviews have suggested that the methodological and reporting quality of ML-based prediction models for clinical outcomes is suboptimal [10–13]. A previously published systematic review of supervised learning models for health care spending had a very narrow scope and did not include a thorough methodological assessment of the literature [14]. In our study, we aim to summarize all ML-based prediction models developed for the prediction of individual-level health care spending, assess their reporting, and appraise their risk of bias. Summarizing the findings of these studies and understanding how they are reported can provide important insights into the strengths and limitations of using ML methods for the prediction of health spending.

## Methods

We designed this systematic review according to the Checklist for Critical Appraisal and Data Extraction for Systematic Reviews of Prediction Modelling Studies (CHARMS) and relevant research guidance by Debray et al. [15, 16]. We report this protocol according to the Preferred Reporting Items for Systematic Reviews and Meta-Analysis Protocols (PRISMA-P) 2015 checklist [17, 18].

### Literature search

We will systematically search PubMed and Embase from inception to 16 September 2021 to identify studies reporting the development, update, or external validation of a prediction model using any ML methods to predict individual-level health care spending or changes in health care spending over any time period, and in any setting.

We will use the following search algorithm: ("machine learning" OR "statistical learning" OR "ensemble" OR "superlearner" OR "transfer learning" OR "classification and regression tree" OR "decision tree" OR "random forest" OR "naive bayes" OR "neural network*" OR "support vector machine" OR "gradient boosting machine" OR "K nearest neighbour" OR "clustering" OR "deep learning" OR "reinforced learning") AND ("high cost*" OR "medical cost*" OR "medical care cost*" OR "health care cost*" OR "healthcare cost*" OR "cost of care" OR "costs of care" OR "per capita cost*" OR "cost bloom" OR "patient spending*" OR "health care spending*" OR "healthcare spending*" OR "medical care spending*" OR "medical spending*" OR "high utilizer*" OR "high need*" OR "super utilizer*" OR "payment*" OR "expenditure*" OR "reimbursement*" OR “risk adjustment”). The set of terms included in the search algorithm are derived from the search terms included in previous systematic reviews of ML-based prediction models for clinical outcomes [10–13]. We will also perform a reference screening of all eligible articles to identify additional studies.

### Eligibility criteria

Table 1 shows a detailed description of the Population, Intervention, Comparator, Outcomes, Timing, and Setting (PICOTS) for this systematic review. The screening of potentially eligible articles will be independently performed by two researchers (AH, MH, NC, LB, OAP). In case of disagreement, consensus will be reached after discussion with all the researchers involved in the screening process. To consider a study as eligible, we will follow the definition of a prediction modelling study as proposed by the Transparent Reporting of a multivariable prediction model for Individual Prognosis or Diagnosis (TRIPOD) statement [19, 20]. Accordingly, a study will be eligible if it reports on the development, update or external validation of a model/algorithm used for predicting an individual’s health care spending as a function of two or more covariates. We will include prediction models that were developed, updated, or validated using ML techniques in patients with any medical condition and in any care setting or time period. We will include models examining binary, continuous, or categorical outcomes relevant to health care costs. We will consider as eligible any observational study (e.g., prospective or retrospective cohort studies and case-control studies), but we will not include any randomized or observational studies designed to evaluate the impact of ML-based prediction models on health care spending.

**Table 1:** Key items for framing aim, search strategy, and study inclusion and exclusion criteria following the PICOTS framework

Item	Definition
Population	Patients with documented costs of health care services in any setting
Intervention	Any prediction model designed to predict individual-level health care spending, patient probabilities for incurring costs of health care services in any setting, or probabilities for any changes in patient costs over time
Comparator	Not applicable
Outcomes	Any cost-related outcome as reported by prediction models
Timing	Predictors measured at any time point preceding outcome; outcome measured in short-term or long-term without applying any specific limitation in prediction horizon
Setting	Any health care setting

We will exclude articles (a) describing ML-based prediction models using ecological data to predict aggregate-level health care spending (e.g., county-level, or country-level); (b) building ML-based models with a primary goal of causal inference, which aim to estimate the change in one’s healthcare costs if a covariate of interest (e.g. insurance) changed from one level (e.g. commercial insurance) to a different level (e.g. public insurance); (c) applying traditional statistical methods, such as linear regression, logistic regression or Cox regression for the prediction purposes; (d) presenting a systematic review of prediction models; (e) describing prediction models using radiomics or speech parameters; (f) building models with biomarkers that are not clinically validated (e.g. genetic polymorphisms), and (g) performing cost-effectiveness analysis without predicting individual-level health care spending. Additionally, we will exclude conference abstracts, because they do not present a detailed description of their methods and their results, which would hinder a thorough methodological assessment. We will also exclude methodological articles that present a novel ML approach for prediction modelling without aiming at building an ML-prediction model for health care spending. Although we will not include systematic reviews as a source of primary data, we will identify any relevant systematic reviews and scrutinize their references to ensure that we include eligible studies that our search algorithm may miss.

We will use version EndNote, version 20 (Clarivate, Philadelphia, Pennsylvania) to perform the deduplication process of the studies retrieved from the literature search. We will use abstrackR for importing citations and performing the title and abstract screening [21].

### Data extraction

To facilitate the data extraction process, we will construct a standardized form by following the CHARMS checklist, previously published research, and relevant recommendations [15, 22–25]. We will use the Systematic Review Data Repository Plus (SRDR+) from the Agency for Healthcare Research & Quality to build the standardized data extraction form and manage the data extraction process, including archiving and sharing data during the review. From each eligible article, we will extract the population characteristics, geographic location, sample size (and number of events for binary outcomes), study design, predicted outcome and its definition, prediction horizon, and measures of model performance (discrimination, calibration, classification, overall performance). We will also extract the ML methods used in the final prediction model, whether the study included development, internal validation, and/or external validation of the model, and whether any model presentation was available in the eligible studies. In the event that an eligible study reports the development of more than one prediction model using ML methods, we will extract information on all reported prediction models. We will specifically evaluate whether the authors reported only apparent performance of a prediction model or examined overfitting by using internal validation. Also, we will examine whether a shrinkage method was applied in eligible studies and which method was used. We will consider that the authors adjusted for optimism sufficiently if they re-evaluated the performance of a model in internal validation and performed shrinkage as well. We will additionally record the data source of predictors, whether there was any inclusion of routinely collected molecular predictors, and whether there were any criteria for manually including or excluding predictors from the final prediction model. Additionally, we will categorize reported validation efforts into categories of internal and external validation [26]. For each eligible study, we also will examine whether the authors reported the presence of missing data on examined outcomes and/or predictors included in the prediction models; if so, we will record how missing data were treated. We will also extract information on how continuous predictors were handled and whether non-linear trends for continuous predictors were assessed.

The data extraction will be independently performed by two researchers (AH, MH, NC, LB, OAP), and disagreements will be resolved after discussion with the rest of the researchers involved in the data extraction process.

### Risk of bias and reproducibility assessment

We will appraise the presence of bias in the studies developing, updating or validating a prediction model by using the Prediction model Risk Of Bias Assessment Tool (PROBAST), which is a risk of bias assessment tool designed for the assessment of diagnostic and prognostic prediction models [27]. It contains multiple questions categorized into four different domains: participants, predictors, outcome, and statistical analysis. Question responses are categorized as either “yes”, “probably yes”, “probably no”, “no”, or “no information”, depending on the characteristics of the study. If a domain contains at least one question signaled as “no” or “probably no”, it is considered high-risk. To be considered low-risk, a domain should contain all questions answered with “yes” or “probably yes”. Overall risk of bias is graded as low-risk when all domains are considered low-risk, and overall risk of bias is considered high-risk when at least one of the domains is considered high-risk.

Moreover, we will appraise the computational reproducibility of the eligible studies by following recently published reproducibility standards [22, 23, 28]. This assessment will be based on the availability of data, models, source codes and dependencies, and analysis plan. We will grade the reproducibility of eligible articles into three categories with varying degrees of rigor for computational reproducibility.

The assessments for risk of bias and reproducibility will be independently performed by two researchers (AH, MH, NC, LB, OAP), and disagreements will be resolved after discussion with the rest of the researchers involved in the assessment process.

## Discussion

As the frequency of applying ML-based prediction models in health economics and outcomes research increases, it is important to track and appraise the quality of studies that report their development in order to facilitate their successful implementation in the real world. To address this need, our systematic review will perform an exhaustive and comprehensive identification, summarization, and synthesis of multivariable models that use ML techniques to predict an individual’s healthcare spending. In addition, we will assess the quality, reporting, and risk of bias of eligible ML-based models and potentially identify models that can be reliably used in the real world. Our findings will summarize the available models and give insights into the strengths and limitations of using ML methods to predict healthcare spending. Through thorough appraisal of the evidence base on ML models for healthcare spending, we will derive recommendations for improving the research practices in prediction modelling of health care spending.

## Data Availability

Not applicable.

## Abbreviations

ML: Machine learning
CHARMS: Checklist for Critical Appraisal and Data Extraction for Systematic Reviews of Prediction Modelling Studies
PICOTS: Population, Intervention, Comparator, Outcomes, Timing, and Setting
TRIPOD: Transparent reporting of a multivariable prediction model for individual prognosis or diagnosis
SRDR+: Systematic Review Data Repository Plus
PROBAST: Prediction model Risk Of Bias Assessment Tool
